# Recessive mutations in the cancer gene *Ataxia Telangiectasia Mutated* (*ATM*), at a locus previously associated with metformin response, cause dysglycaemia and insulin resistance

**DOI:** 10.1111/dme.13037

**Published:** 2015-12-24

**Authors:** P. J. Connelly, N. Smith, R. Chadwick, A. R. Exley, J. M. Shneerson, E. R. Pearson

**Affiliations:** ^1^ Division of Cardiovascular and Diabetes MedicineMedical Research InstituteUniversity of DundeeDundeeUK; ^2^ Papworth Hospital NHS Foundation TrustCambridgeUK

## Abstract

**Aim:**

To investigate glucose and insulin metabolism in participants with ataxia telangiectasia in the absence of a diagnosis of diabetes.

**Methods:**

A standard oral glucose tolerance test was performed in participants with ataxia telangiectasia (*n* = 10) and in a control cohort (*n* = 10). Serial glucose and insulin measurements were taken to permit cohort comparisons of glucose‐insulin homeostasis and indices of insulin secretion and sensitivity.

**Results:**

During the oral glucose tolerance test, the 2‐h glucose (6.75 vs 4.93 mmol/l; *P* = 0.029), insulin concentrations (285.6 vs 148.5 pmol/l; *P* = 0.043), incremental area under the curve for glucose (314 vs 161 mmol/l/min; *P* = 0.036) and incremental area under the curve for insulin (37,720 vs 18,080 pmol/l/min; *P* = 0.03) were higher in participants with ataxia telangiectasia than in the controls. There were no significant differences between groups in fasting glucose, insulin concentrations or insulinogenic index measurement (0.94 vs 0.95; *P* = 0.95). The Matsuda index, reflecting whole‐body insulin sensitivity, was lower in participants with ataxia telangiectasia (5.96 vs 11.03; *P* = 0.019) than in control subjects.

**Conclusions:**

Mutations in *Ataxia Telangiectasia Mutated (ATM)* that cause ataxia telangiectasia are associated with elevated glycaemia and low insulin sensitivity in participants without diabetes. This indicates a role of *ATM* in glucose and insulin metabolic pathways.

## What's new?

- In a genome‐wide association study in 2011, our group identified *Ataxia Telangiectasia Mutated (ATM*) as a potential candidate gene associated with glycaemic response to metformin.
- In this study, we investigate the role of *ATM* in carbohydrate metabolism by performing an oral glucose tolerance test in participants with recessive mutations in this gene, causing the condition ataxia telangiectasia, and healthy control subjects.
- We show that ataxia telangiectasia is associated with elevated glycaemia and decreased insulin sensitivity.
- These results indicate a significant role of *ATM* in glucose and insulin metabolic pathways.

**What's new?**

- In a genome‐wide association study in 2011, our group identified *Ataxia Telangiectasia Mutated (ATM*) as a potential candidate gene associated with glycaemic response to metformin.
- In this study, we investigate the role of *ATM* in carbohydrate metabolism by performing an oral glucose tolerance test in participants with recessive mutations in this gene, causing the condition ataxia telangiectasia, and healthy control subjects.
- We show that ataxia telangiectasia is associated with elevated glycaemia and decreased insulin sensitivity.
- These results indicate a significant role of *ATM* in glucose and insulin metabolic pathways.

## Introduction

The anti‐hyperglycaemic agent, metformin, is recommended as the first‐line oral therapy for the treatment of Type 2 diabetes in both national and international guidelines; however, despite being widely prescribed for >50 years, the mechanisms underpinning metformin's actions and the considerable variation in response to this drug are only now emerging 1, 2.

Recently, we reported in a genome‐wide study an association between the single‐nucleotide polymorphism (SNP) rs11212617 and metformin glycaemic response 3. This SNP is located on chromosome 11 within a large linkage disequilibrium block that includes a total of seven potential candidate genes. As with any genome‐wide association study, additional functional, mouse and human studies are required to confirm the metformin response variant at this locus.

A potential causal gene responsible for this relationship is *Ataxia Telangiectasia Mutated* (*ATM*)*,* a serine/threonine protein kinase with a central role in the cellular response to DNA damage 4. Although this cancer gene has not previously been reported to be associated with metformin action, there is growing evidence of the role of *ATM* in glucose metabolism.

It has been shown that *ATM‐*deficient mice exhibit glucose intolerance, insulin resistance and impaired insulin secretion 5, 6, while interactions of this protein kinase with multiple components of insulin signalling (JNK, PI3K, IRS2 and AKT) have been observed in *in vitro* studies 7. Furthermore, case reports from the 1970s in a limited number of participants with diabetes and loss of functional *ATM* mutations, resulting in the autosomal recessive condition ataxia telangiectasia, showed marked insulin resistance 8.

Given these previous case reports of insulin resistance in ataxia telangiectasia, and the novel finding that *ATM* may influence metformin response, we aimed to investigate glucose metabolism in participants with ataxia telangiectasia in the absence of a diagnosis of diabetes.

## Participants and methods

### Participants

Participants with ataxia telangiectasia (*n* = 10) were recruited from the National Adult Ataxia Telangiectasia Centre, Papworth Hospital, in collaboration with the Ataxia Telangiectasia Society. Participants with ataxia telangiectasia were aged >16 years, non‐pregnant and formally diagnosed with ataxia telangiectasia. The control subjects (*n* = 10) were identified from the UK Type 2 Diabetes Case Control collection study and were matched for age, sex and BMI. All participants were white. This study was granted ethical approval by the East of Scotland Research Ethics Committee and informed consent was obtained from all participants.

### Methods

The study participants attended Papworth Hospital, Cambridge or Ninewells Hospital, Dundee. Participants fasted overnight and underwent a standard 75‐g oral glucose tolerance test (OGTT). Glucose and insulin samples were collected in fluoride and lithium‐heparin tubes respectively at 0, 30, 60, 90 and 120 min. Samples were centrifuged immediately and stored at −80°C. Glucose concentrations were analysed using the hexokinase method with Siemens ADVIA 2400 (Siemens Healthcare Diagnostics Inc, Tarrytown, NY, USA). Insulin was measured using the AutoDELFIA assay (PerkinElmer, Waltham, MA, USA).

### Calculations

The incremental area under the curve (AUC) of plasma glucose and insulin during the OGTT were calculated according to the trapezoid rule and reported to the nearest integer. Insulin secretion was evaluated by calculating the AUC of insulin to AUC of glucose ratio (AUC_ins/glu_). The Matsuda index was calculated as an approximation of composite whole‐body insulin sensitivity 9. Homeostasis model assessment of insulin resistance (HOMA‐IR) values were calculated by dividing a multiple of fasting insulin (mU/l) and glucose (mmol/l) concentrations by 22.5. The insulinogenic index and homeostasis model assessment of β‐cell function (HOMA‐β) were calculated as indices of β‐cell function. Insulinogenic index was calculated as Δinsulin (mU/ml)/Δglucose (mg/dl) between 0 and 30 min, and HOMA‐β was calculated as: 20 × fasting glucose (mmol/l) divided by [fasting insulin (mU/l)‐3.5]. When required for calculations, glucose and insulin were converted to mg/dl and mU/l by a conversion factor of 0.0555 and 6.0 respectively.

### Statistical analysis

Analysis was performed using stata version 10.1 (Stata Corp., College Station, TX, USA). Our study had 80% power (α = 0.05) to detect the observed differences in our primary outcome, insulin sensitivity. Two‐tailed unpaired *t*‐tests were used for comparisons between groups. Welch's correction was used for comparisons with unequal variances. The Mann*–*Whitney *U*‐test was used for non‐parametric data. A two‐sided *P* value <0.05 was taken to indicate statistical significance. Data are presented as means (sem).

## Results

**Figure 1:**
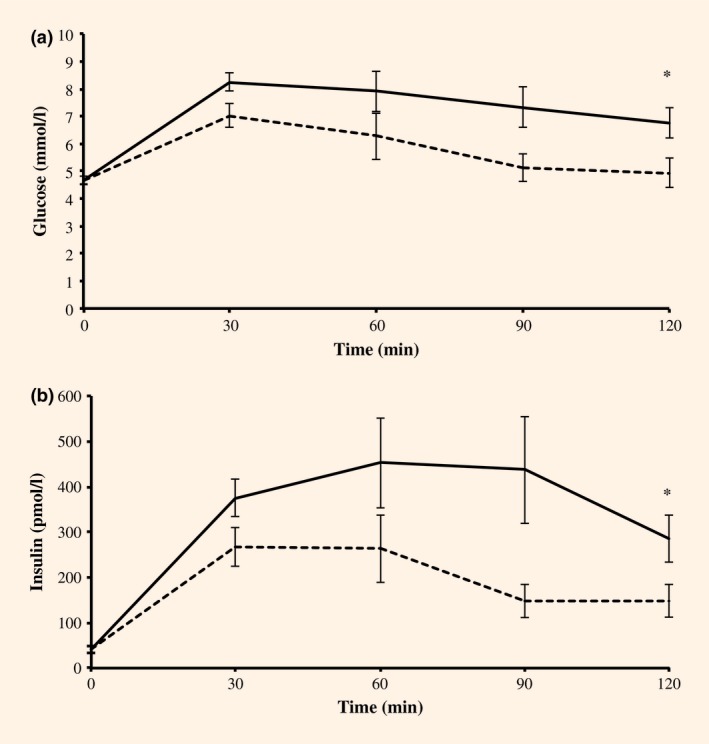
Glucose (a) and insulin (b) excursions during a 2‐h OGTT. Mean values ± sem for the ataxia telangectasia (solid line) and control (dashed line) cohorts are charted. *P < 0.05.

Participants with ataxia telangiectasia and control participants were well matched for age, sex and BMI (Table 1). The mean fasting glucose (Fig. 1) for participants with ataxia telangiectasia was 4.68 mmol/l compared with 4.65 mmol/l in control subjects (*P* = 0.89; 95% CI 0.42, 0.48). In participants with ataxia telangiectasia 2‐h glucose concentrations were 6.75 mmol/l vs 4.93 mmol/l in control subjects (*P* = 0.029; 95% CI 0.21, 3.42).

**Table 1:** Participant characteristics, glucose‐insulin homeostasis variables and insulin sensitivity indices in the ataxia telangiectasia and control cohorts

	Ataxia telangiectasia	Control	*P*
Age, years	39.4 ± 2.7	38.9 ± 2.3	–
Men, *n* (%)	4 (40)	4 (40)	–
BMI, kg/m^2^	24.7 ± 1.8	25.2 ± 2.5	–
Glucose–insulin homeostasis			
Incremental 2‐h glucose AUC, mmol/l/min	314 ± 57.3	161 ± 36.4	0.036
Incremental 2‐h AUC insulin, pmol/l/min	37,720 ± 7,424	18,080 ± 3,496	0.03
AUC_ins/glu_ ratio	132.0 ± 107.4	159.9 ± 97.2	0.19
Indices of insulin sensitivity and secretion			
HOMA‐β	64.42 ± 73.39	112.47 ± 67.52	0.56
Insulinogenic index	0.94 ± 0.13	0.95 ± 0.12	0.95
Matsuda index	5.96 ± 0.77	11.03 ± 1.69	0.019
HOMA‐IR	1.41 ± 0.23	1.50 ± 0.35	0.81

AUC, area under the curve; AUC_ins/glu_ ratio, AUC of insulin to AUC of glucose ratio; HOMA‐ β, homeostasis model assessment of β‐cell function; HOMA‐IR, homeostasis model assessment of insulin resistance.

Data are expressed as means ± sem.

Fasting insulin concentrations were 39.7 and 41.4 pmol/l in the ataxia telangiectasia and control groups, respectively (*P* = 0.87). The 2‐h insulin concentrations were higher in the ataxia telangiectasia group than in the control group (mean difference=137.1 pmol/l; *P* = 0.043; 95% CI 4.4, 269.8). Mean incremental AUC glucose was significantly higher in the ataxia telangiectasia group compared with the control group with a mean difference of 153 mmol/l/min (*P* = 0.036; 95% CI 10.73, 295.87). Mean incremental AUC insulin was also higher in the ataxia telangiectasia group with a mean difference of 19 640 pmol/l/min (*P* = 0.03; 95% CI 1997.3, 32,783.6). The AUC_ins/glu_ ratio was 132.0 in the ataxia telangiectasia group and 159.9 in the control group, with a mean difference of 27.9 (*P* = 0.19).

There were no significant differences between HOMA‐β (*P* = 0.56) and insulinogenic index (*P* = 0.95) as indices of β‐cell insulin secretion. HOMA‐IR also did not differ between the two groups (*P* = 0.81). The Matsuda index was higher, however, in participants with ataxia telangiectasia than in the control subjects (5.96 vs 11.03), with a mean difference of 5.1 (*P* = 0.019; 95% CI −9.13, −1.01) consistent with insulin resistance in the ataxia telangiectasia group.

## Discussion

This study supports the role of *ATM* mutations in abnormal glucose tolerance. Following a 2‐h OGTT, incremental AUC glucose values were ~95% higher in participants with ataxia telangiectasia compared with control subjects, implying significant postprandial hyperglycaemia. Similarly, there was a marked but appropriate increase in insulin concentrations in participants with ataxia telangiectasia after the glucose challenge.

Interestingly, there was no difference in insulinogenic index or HOMA‐β as surrogate markers of β‐cell function, but rather a reduction in whole‐body composite insulin sensitivity. As HOMA‐IR values were equivalent between groups, such results probably represent a reduction in peripheral, rather than hepatic, insulin sensitivity. This observation is at odds with the relationship between ATM and metformin response, as this drug predominantly acts on the liver 1, 2; however, our understanding of metformin pharmacology is ever‐changing and ATM substrates may be implicated in the wider anti‐hyperglycaemic role of this drug.

The insulin excursions observed in participants with ataxia telangiectasia in the present study were modest compared with previous reports of participants with ataxia telangiectasia and diabetes 8. This difference may represent either a less severe phenotype, as our participants did not have diabetes, or the historical use of non‐specific insulin radioimmunoassays that were prone to cross‐reactivity with insulin‐like molecules. Nevertheless, our results show a relative reduction in insulin sensitivity in participants with ataxia telangiectasia when compared with healthy participants. People with Ataxia telangiectasia are reported to have an increased risk of developing diabetes 10. Our results are consistent with this being largely mediated via an insulin‐resistant mechanism.

One weakness of the present study is that an OGTT was undertaken rather than a euglycaemic clamp study because of the challenges of performing the latter in physically impaired participants. Although the indices obtained from an OGTT are estimates, they correlate well with results obtained from clamp studies 9, 11. This weakness could be addressed by performing clamp studies in obligate heterozygous parents of participants with ataxia telangiectasia, which would also permit the study of metformin response directly.

The dysglycaemic ataxia telangiectasia phenotype suggests that ATM may regulate several processes important in carbohydrate metabolism. ATM has been shown to participate in insulin signalling via phosphorylation of elF‐4E‐binding protein 1^4^. Furthermore, this kinase mediates the full activation of AKT activity, regulates GLUT4 translocation by insulin in skeletal muscle, and may even regulate adipocyte differentiation, which may in part explain the postprandial dysglycaemia evident in people with ataxia telangiectasia 12, 13.

Our results are highly relevant to a number of disease processes. People with ataxia telangiectasia and those with germline *ATM* mutations are predisposed to cancer, while somatic *ATM* mutations and perturbed ATM signalling promote tumourgenesis 14. In addition to ATM's role in DNA damage response, glucose restriction, as a consequence of ATM deficiency, may be advantageous to the survival of solid tumours through processes such as autophagy. Furthermore, variation in the *ATM* gene has been associated with coronary artery disease 15. Such observations suggest a potential role of ATM within the development of metabolic disease, possibly through alterations in insulin metabolism and resistance.

In summary, the present study shows that participants possessing recessive loss of function mutations in *ATM* have dysglycaemia and lower insulin sensitivity when compared with a control cohort. This indicates that *ATM* has a role in glucose and insulin metabolic pathways. Further studies are required to characterize the mechanisms responsible for such interactions.

## Funding sources

This study was funded by The Anonymous Trust and supported by the Wellcome Trust [102820/Z/13/Z].

## Competing interests

None declared.
